# Malonylation of histone H2A at lysine 119 inhibits Bub1-dependent H2A phosphorylation and chromosomal localization of shugoshin proteins

**DOI:** 10.1038/s41598-018-26114-z

**Published:** 2018-05-16

**Authors:** Tadashi Ishiguro, Kana Tanabe, Yuki Kobayashi, Shinsuke Mizumoto, Motomu Kanai, Shigehiro A. Kawashima

**Affiliations:** 1 Graduate School of Pharmaceutical Sciences, The University of Tokyo, 7-3-1 Hongo, Bunkyo-ku, Tokyo, 113-0033 Japan; 2 JST-ERATO, Kanai Life Science Catalysis Project, 7-3-1, Hongo, Bunkyo-ku, Tokyo, 113-0033 Japan

## Abstract

Post-translational modifications of histones, such as acetylation and phosphorylation, are highly conserved in eukaryotes and their combination enables precise regulation of many cellular functions. Recent studies using mass spectrometry have revealed various non-acetyl acylations in histones, including malonylation and succinylation, which change the positive charge of lysine into a negative one. However, the molecular function of histone malonylation or succinylation is poorly understood. Here, we discovered the functions of malonylation in histone H2A at lysine 119 (H2A-K119) in chromosome segregation during mitosis and meiosis. Analyses of H2A-K119 mutants in *Saccharomyces cerevisiae* and *Schizosaccharomyces pombe* showed that anionic mutations, specifically to aspartate (K119D) and glutamate (K119E), showed mis-segregation of the chromosomes and sensitivity to microtubule-destabilizing reagents in mitosis and meiosis. We found that the chromosomal localization of shugoshin proteins, which depends on Bub1-catalyzed phosphorylation of H2A at serine 121 (H2A-S121), was significantly reduced in the H2A-K119D and the H2A-K119E mutants. Biochemical analyses using K119-unmodified or -malonylated H2A-C-tail peptides showed that H2A-K119 malonylation inhibited the interaction between Bub1 and H2A, leading to a decrease in Bub1-dependent H2A-S121 phosphorylation. Our results indicate a novel crosstalk between lysine malonylation and serine/threonine phosphorylation, which may be important for fine-tuning chromatin functions such as chromosome segregation.

## Introduction

Chromosomes are composed of nucleosomes, which contain two copies of the histone proteins H2A, H2B, H3, and H4, and DNA. A large number of residues within the histones are covalently modified by post-translational modifications (PTMs), such as acetylation, methylation, ubiquitination, and phosphorylation. Each PTM in histones has specific functions in diverse cellular processes, including gene transcription, DNA replication and repair, recombination, mitosis, and meiosis. For example, histone acetylation is generally linked to gene expression^1^. Acetylation of lysine residues switches the charge state of lysine residues from cationic to neutral, which promotes chromatin decompaction and gene transcription. In addition, reader proteins recognize the lysine acetylation in histones and promote gene transcription. On the other hand, histone phosphorylation is involved in not only gene transcription, but also chromosome condensation and segregation during mitosis and meiosis^2^. It is increasingly clear that these histone PTMs regulate one another, providing regulatory crosstalk^3^. One PTM affects the occurrence of subsequent PTMs, either on the same histone molecule (*in cis*) or between histone molecules (*in trans*), which enables precise regulation of many cellular functions.

Recent studies using high performance liquid chromatography – tandem mass spectrometry (LC-MS/MS) have uncovered various non-acetyl acylations in histones^4,5^. So far, three types of non-acetyl acylations have been identified in histones. First, hydrophobic acylation, such as propionylation^6^, butyrylation^6^, and crotonylation^7^, neutralizes the positive charges of lysine residues. Second, acidic acylation, which encompasses malonylation^8^, succinylation^8^, and glutarylation^9^, turns the positive charge of lysine residues into a negative one. Third, hydroxylated acylation, which refers to 2-hydroxyisobutyrylation^10^ and β-hydroxybutyrylation^11^, can allow the formation of hydrogen bonds with other molecules. Recent studies have suggested that hydrophobic acylation and hydroxylated acylation in histones are linked to active gene transcription, just like acetylation^10–14^. In contrast, the function of acidic acyl groups in histones is poorly understood. Moreover, crosstalk between non-acetyl acylations and other PTMs has not been reported yet.

**Figure 1:**
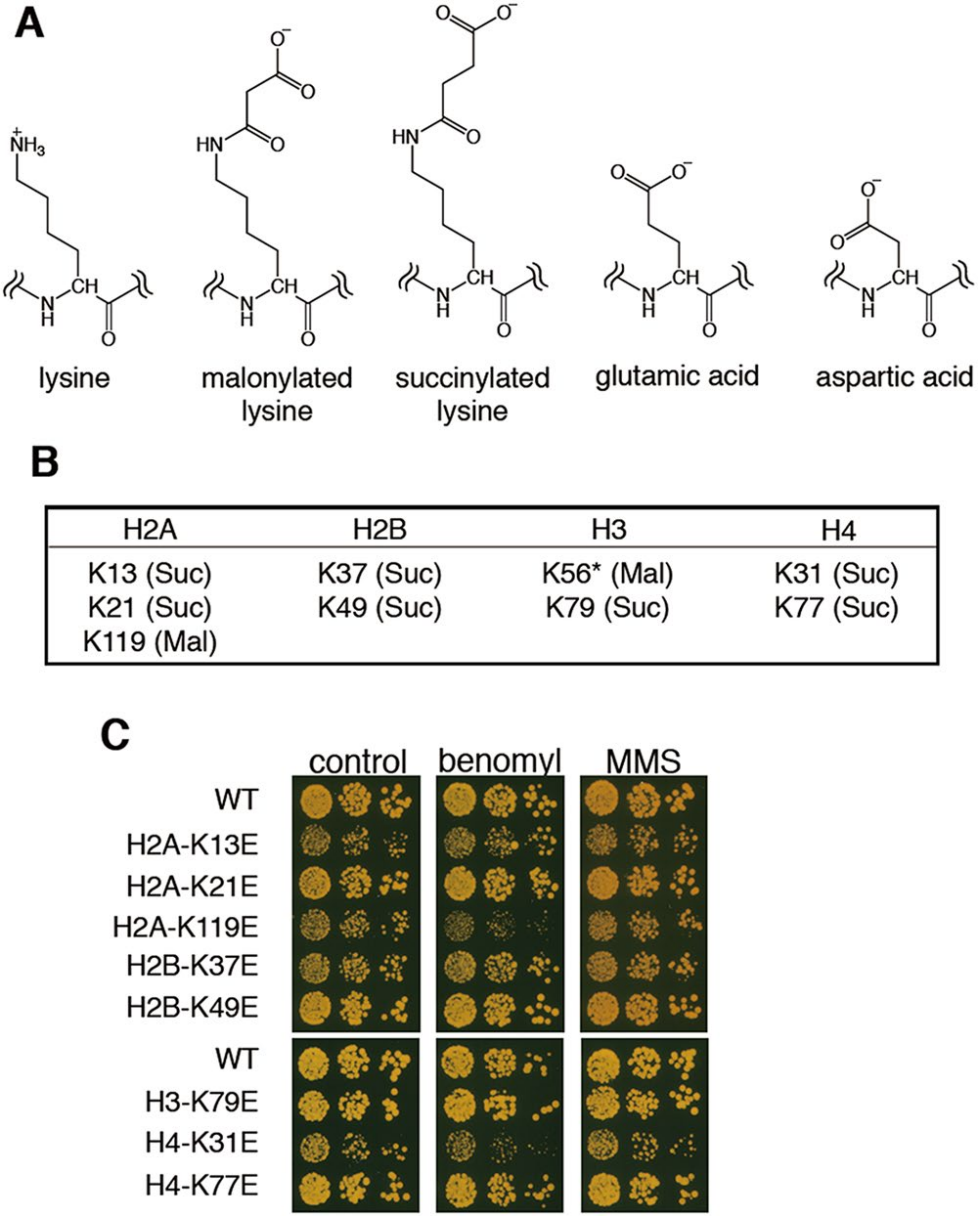
Glutamic acid mutant of H2A-K119, a malonylation site in budding yeast, shows specific benomyl sensitivity. (**A**) Chemical structures of unmodified lysine, malonylated lysine, succinylated lysine, glutamic acid, and aspartic acid. Under neutral aqueous conditions, the side chain of unmodified lysine exhibits a cationic charge; on the other hand, the side chains of malonylated or succinylated lysine, glutamic acid, and aspartic acid exhibit an anionic charge by ionization. (**B**) Lysine residues that have been reported to be malonylated (Mal) or succinylated (Suc) in *Saccharomyces cerevisiae*. *H3K56E mutation is lethal. (**C**) Serial dilutions (1000, 200, 40 cells) of WT and indicated histone mutant strains of budding yeast were grown on YPD plates or YPD plates containing benomyl 15 µg/ml) or methylmethanesulfonate (MMS, 0.016%), and incubated at 29 °C for 2 days.

Lysine succinylation and lysine malonylation (Fig. 1A) were originally discovered in non-histone proteins^15,16^, affording some insights about their effects. The succinylation of carbamoyl phosphatase synthase (CPS1) regulates its enzymatic activity^17^, while the malonylation in glyceraldehyde 3-phosphate dehydrogenase (GAPDH) is important for its enzymatic activity^18^. The following study described identification of these two PTMs in histones in several organisms, including *Saccharomyces cerevisiae* (budding yeast)^8^. Proteomic profiling studies have shown that proteins that undergo malonylation are typically different from the proteins undergoing acetylation or succinylation^18,19^, suggesting that histone succinylation and malonylation are regulated by distinct mechanisms. Removal of both succinylation and malonylation is catalyzed by Sirt5, an isoform of the sirtuin family of lysine deacylase^17^. One very recent study has revealed lysine acetyltransferase 2A (KAT2A, also known as Gcn5) to be a succinyltransferase^20^, whereas enzymes that catalyze lysine malonylation have not been identified to date.

In this study, we constructed and examined a series of malonyl- or succinyl-mimetic budding yeast mutants, and found the functions of malonylation in histone H2A at lysine 119 (H2A-K119) in chromosome segregation during mitosis and meiosis. H2A-K119 malonylation inhibits the interaction between Bub1 and H2A, leading to the loss of Bub1-dependent phosphorylation of H2A-S121, and consequently the failure of shugoshin proteins to localize to the centromere.

## Results

### Examining malonyl- or succinyl-mimetic mutants in budding yeast

We sought to address whether some instances of histone malonylation or succinylation were linked to chromosome segregation mechanisms during mitosis. Since the anionic character of malonylated or succinylated lysine can be partly mimicked by the mutation of lysine to glutamic acid or aspartic acid (Fig. 1A), we used plasmid shuffling to construct budding yeast histone mutants, in which a lysine residue was mutated to glutamic acid (Fig. S1A). In this study, we examined nine lysine residues that have been reported to be malonylated or succinylated in *Saccharomyces cerevisiae* (budding yeast) (Fig. 1A)^8^. H3K56E was excluded from our analysis because of its lethality^8^. Among the mutants we tested, only the H2A-K119E and H4K31E mutants showed sensitivity to benomyl, a microtubule depolymerizing agent which often enhances chromosome segregation defects, whereas no mutants were significantly sensitive to methylmethanesulfonate (MMS), a DNA-damaging agent (Fig. 1C). We decided to focus on the analysis of H2A-K119, since phosphorylation at proximal serine 121 (H2A-S121) is essential for the chromosomal localization of shugoshin (Sgo1) protein that ensures faithful chromosome segregation^21^.

### H2A-K119 malonyl-mimetic mutants cause chromosome segregation defects in budding yeast

**Figure 2:**
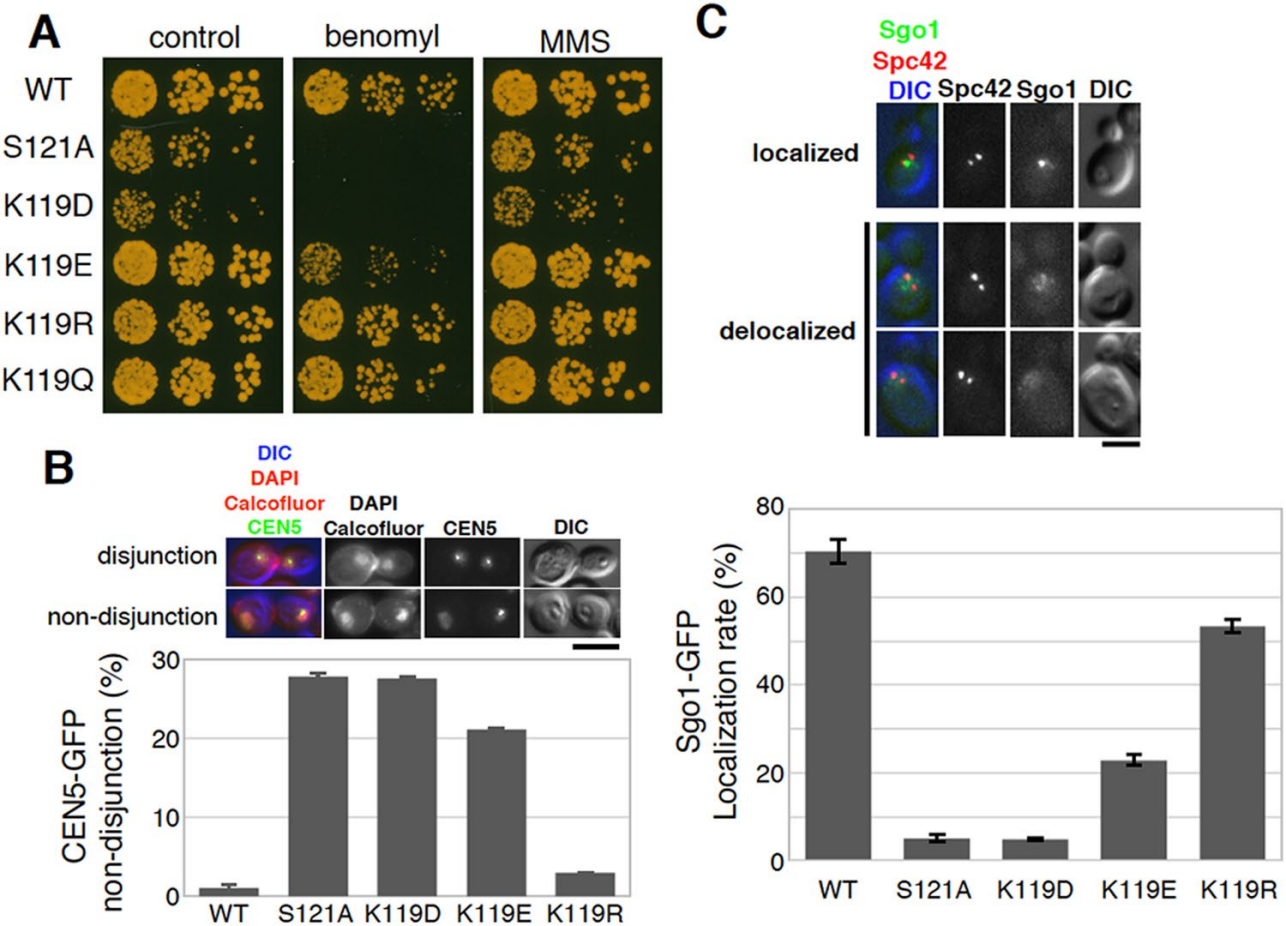
H2A-K119 malonyl-mimetic mutations lead to chromosome mis-segregation and loss of shugoshin localization in budding yeast. (**A**) Serial dilutions (1000, 200, 40 cells) of WT or indicated H2A mutant cells of budding yeast were spotted onto YPD plates or YPD plates containing benomyl (15 µg/ml), and incubated at 29 °C for 2 days. (**B**) The patterns of *CEN5*-GFP in separated nuclei in mother and daughter cells still linked after mitotic anaphase were sorted into the categories ‘disjunction’ and ‘non-disjunction,’ as shown in the upper panel. Cell pairs were determined by DIC and Calcofluor staining. The rate of non-disjunction in each strain grown in YPD containing 10 µg/mL benomyl was quantified in the lower graph (n > 90, error bar = range of 2 experiments, scale bar = 5 µm). (**C**) The patterns of Sgo1-GFP localization in mitotic metaphase cells were judged as ‘localized’ or ‘delocalized,’ as shown in the upper panel. SPB separation, monitored by Spc42-mCherry signal, was used to choose metaphase cells. Quantification data is shown in the lower graph. (n > 100, error bar = range of 2 experiments, scale bar = 3 µm).

In order to examine whether the anionic property of H2A-K119 induced benomyl sensitivity, we further constructed several H2A-K119 mutants, including an aspartic acid mutant K119D as another malonyl-mimetic mutant, a glutamine mutant K119Q that neutralizes lysine’s positive charge, and an arginine mutant K119R that keeps the positive charge. Immunoblotting experiments of yeast cell extracts showed that H2A mutants expressed at level comparable to the wild-type protein (Fig. S2A). Intriguingly, the K119D and K119E mutants showed benomyl sensitivity, whereas K119R and K119Q did not (Figs 2A, S2B). Consistently, we observed a high frequency of chromosome mis-segregation in benomyl-treated K119D and K119E mutants, but not in the benomyl-treated WT or K119R mutant (Figs 2B, S2B). As the H2A-S121A mutant, in which serine 121 of H2A was substituted with a non-phosphorylatable alanine, showed benomyl sensitivity and chromosome mis-segregation with a tendency similar to the K119D mutant, we hypothesized that H2A-K119 malonylation affected phosphorylation at H2A-S121. To address the hypothesis, we observed the centromeric localization of Sgo1-GFP in metaphase cells, which requires H2A-S121 phosphorylation^21^. Compared to the wild-type, Sgo1-GFP localization was dramatically reduced in the H2A-K119D and K119E mutants, as was observed in the H2A-S121A mutant (Fig. 2C). On the other hand, Sgo1-GFP localization in the K119R mutant was only slightly reduced (Fig. 2C). These data suggest that the negative charge at H2A-K119 may inhibit the H2A-S121 phosphorylation that is required for chromosomal Sgo1 localization, and thus induce chromosome mis-segregation during mitosis.

### The effects of H2A-K119 malonyl-mimetic mutation are conserved in fission yeast

**Figure 3:**
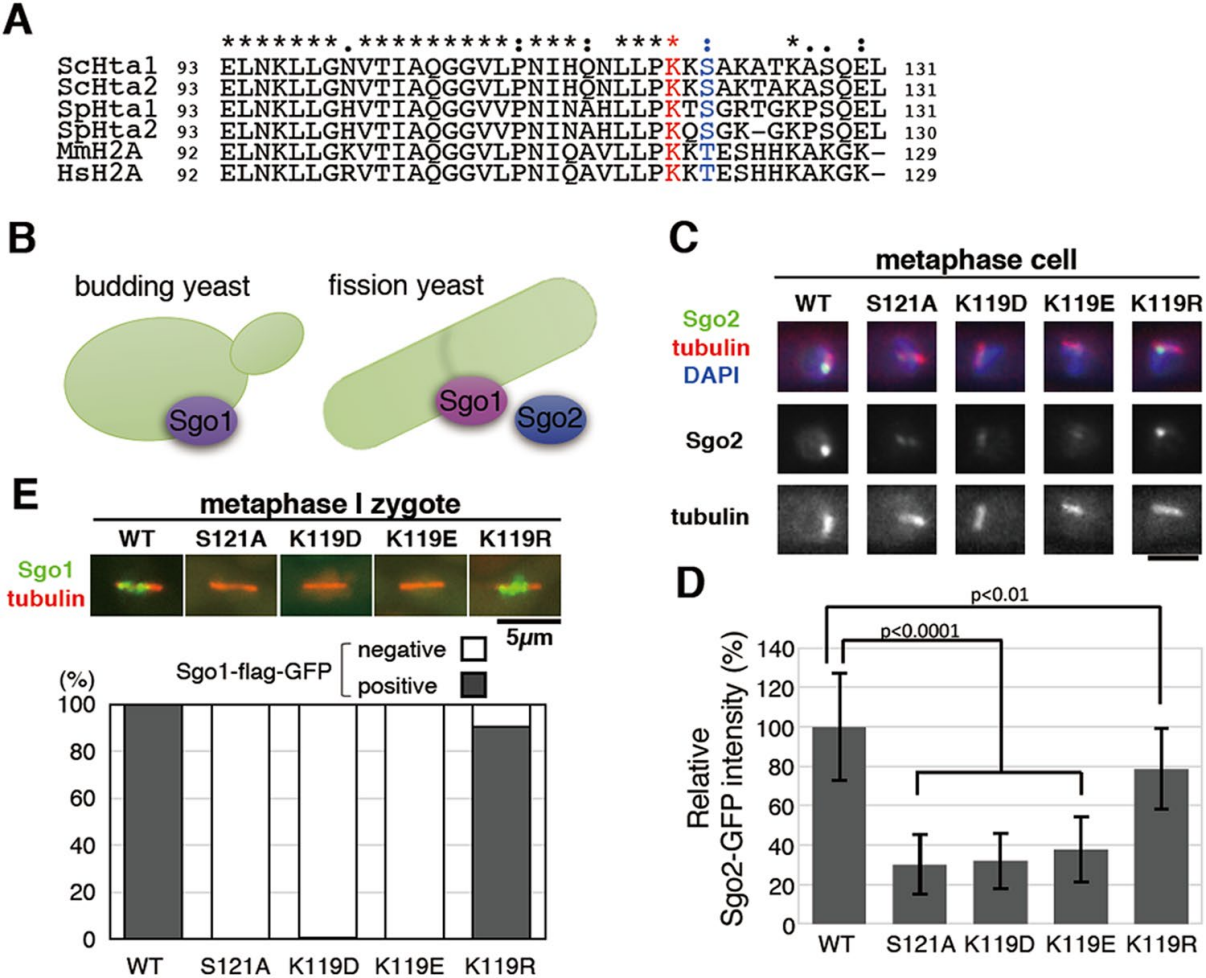
Both shugoshin homologs fail to localize to chromosomes in fission yeast H2A-K119 malonyl-mimetic mutants. (**A**) The alignment of amino acid seqences of the H2A-C-tail in several H2A orthologs. Sc, *Saccharomyces serevisiae*; Sp, *Schizosaccharomyces pombe*; Mm, *Mus musculus*; Hs, *Homo sapiens*. The residue identical to K119 of budding yeast is marked in red. The residue identical to S121 of budding yeast is in blue. (**B**) Budding yeast has a sole shugoshin homolog Sgo1, whereas fission yeast has the two homologs Sgo1 and Sgo2. (**C**) Representative images of Sgo2-GFP and mitotic metaphase spindle in wild type and indicated mutant strains of fission yeast. For quantification, cells with short metaphase spindle (1–2 µm) were selected. scale bar = 2.5 µm. (**D**) Signal intensity of Sgo2-GFP at mitotic metaphase observed in (**C**) was measured. (n = 20, error bar = SD). Unpaired two-tailed Student’s t test significance values were shown. (**E**) Wild-type or mutant H2A zygotes of fission yeast expressing Sgo1-flag-GFP were arrested at metaphase I by repressing *slp1* and *cut23*. Upper panel shows representative images of centromeric Sgo1-flag-GFP and meiotic metaphase I spindle in each strain. The lower graph shows the percent of cells negative (white) or positive (black) for Sgo1-flag-GFP localization, in zygotes with meiotic metaphase I spindle (n > 120), by strain.

Since H2A-K119 is widely conserved among several eukaryotes including fission yeast (Fig. 3A), we next constructed H2A-K119 mutants in fission yeast (Fig. S1B) and examined their phenotypes. ChIP (Chromatin immunoprecipitation) analysis showed that H2A mutants deposited at the centromeres as well as the wild-type H2A (Fig. S3A). We found that malonyl-mimetic K119D and K119E mutants showed sensitivity to a microtubule depolymerizing agent (TBZ) in the same way the H2A-S121A mutant had (Fig. S3B), suggesting that H2A-K119-related mechanisms are conserved between the two yeasts. In contrast, the H2A-K119R mutant showed weaker TBZ-sensitivity than these mutants. We next addressed whether H2A-K119 malonyl-mimetic mutations affected chromosomal localization of shugoshin proteins in fission yeast. In contrast to budding yeast that has only shugoshin Sgo1, fission yeast has two shugoshin paralogs, Sgo1 and Sgo2 (Fig. 3B)^22,23^. Fission yeast Sgo1 is transiently expressed during the first meiotic division (meiosis I) and maintains centromeric cohesion through the recruitment of the protein phosphatase 2A complex^22,24^, whereas Sgo2 is expressed during both mitotic and meiotic divisions and ensures proper attachment between the kinetochores and microtubules by recruiting the Aurora B kinase complex^23,25^. Intriguingly, we found that both Sgo2 centromeric localization during mitosis and Sgo1 centromeric localization during meiosis I were dramatically delocalized in the K119D and K119E mutants as well as the H2A-S121A mutant (Fig. 3C–E). The lysine to arginine mutation (K119R) did not significantly affect shugoshin localization (Fig. 3C–E). The mutation at H2A-K119 may not affect protein level of shugoshin proteins, since the level of Sgo2-GFP in any H2A mutants was comparable to the wild-type protein (Fig. S3C). Centromeric Sgo1 is essential for the protection of sister chromatid cohesion during meiosis I, so Sgo1 delocalization leads to the non-disjunction of sister chromatids at the second meiotic division (meiosis II)^21,22^. Consistently, sister chromatid non-disjunction at meiosis II was significantly increased in the K119D and K119E mutants as well as the H2A-S121A mutant (Fig. S3D). These results suggest that H2A-K119 malonylation-mediated inhibition of shugoshin localization is likely conserved between budding yeast and fission yeast.

### H2A-K119 malonylation inhibits H2A-S121 phosphorylation by Bub1

**Figure 4:**
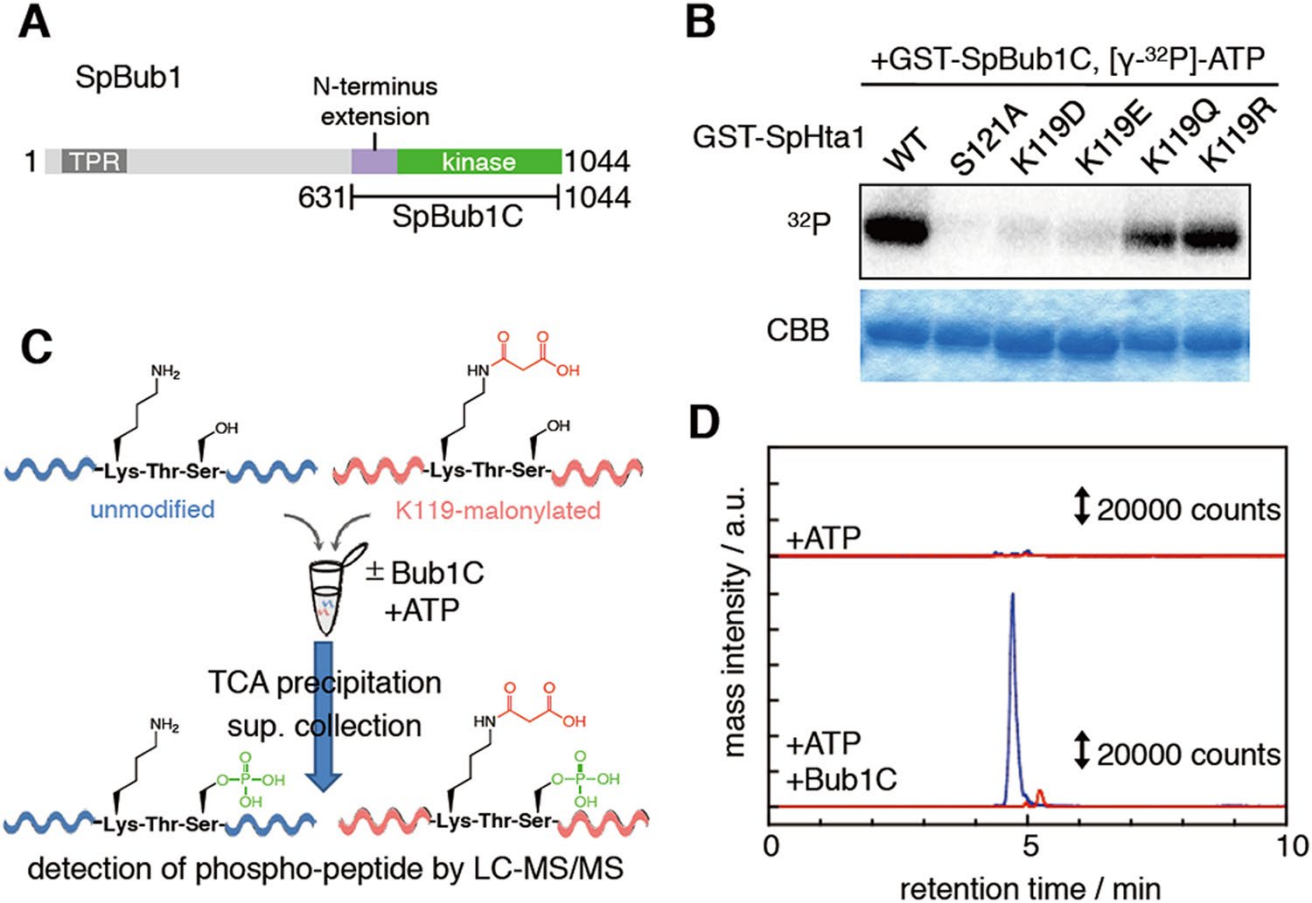
H2A-K119 malonylation inhibits Bub1-dependent phosphorylation of H2A. (**A**) Schematic of fission yeast’s Bub1. TPR, tetratricopeptide repeat; kinase, kinase domain. *C*-terminus fragment (SpBub1C) were prepared for further analysis. (**B**) Recombinant GST-SpHta1 proteins of wild type or indicated mutants were incubated with recombinant GST-SpBub1C in the presence of [γ-^32^P]-ATP. The incorporation of ^32^P was detected by autoradiography. Protein loading amount was checked by Coomassie Brilliant Blue (CBB) staining. Uncropped images are shown in Fig. S6. (**C**) Schematic work flow of peptide phosphorylation assay. Equal amounts of synthesized control peptide (VPNINAHLLPKTSGRTGK) and K119 malonylated peptide (VPNINAHLLPK_mal_TSGRTGK) were mixed, phosphorylated by GST-SpBub1C, and purified. The S121-phosphorylated individual peptides were detected by LC–MS/MS. (**D**) LC chromatograms in LC-MS/MS analysis of S121 phosphorylated peptide obtained from control peptide (blue) and K119 malonylated peptide (red). Precursor ions were detected as [*M* + 4H]^4+^ (*m*/*z* = 496.27 (pS121), 517.27 (pS121, K119mal)).

As H2A-S121 phosphorylation is catalyzed by Bub1 kinase^21^, we addressed whether malonyl-mimetic mutations inhibited H2A phosphorylation by Bub1 *in vitro*. For this purpose, we purified fission yeast’s recombinant Bub1C protein (SpBub1C), made up of the kinase domain and the N-terminus extension, which has been reported to be important for the kinase activity^26^ of fission yeast Bub1 (Fig. 4A). The recombinant proteins of fission yeast H2A (SpHta1) were also purified. An *in vitro* kinase assay using these recombinant proteins showed that the H2A-K119D and the H2A-K119E mutants, as well as the H2A-S121A mutant, were not significantly phosphorylated by Bub1, whereas H2A-K119R mutant proteins were phosphorylated by Bub1 (Fig. 4B). An acetyl-mimetic H2A-K119Q mutation slightly inhibited Bub1-mediated H2A phosphorylation (Fig. 4B). We repeated the *in vitro* kinase assay using budding yeast’s H2A (ScHta1) and Bub1 kinase (ScBub1C) proteins and obtained similar results (Fig. S4A,B). Therefore, we concluded that the K119 malonyl-mimetic mutation inhibits H2A-S121 phosphorylation by Bub1.

Although the effects of H2A-K119 malonyl-mimetic mutations are now clearer, it might be possible that malonyl-mimetic mutations do not completely mimic malonylated lysine. Therefore, we next sought to address the effects of true malonylation at H2A-K119 on H2A-S121 phosphorylation by Bub1. For this purpose, we synthesized H2A C-tail peptides (V109–K126 of SpHta1) with and without malonylation at K119. The mixture of both peptides was reacted with or without SpBub1C in the presence of ATP, and the phosphorylated peptides were analyzed by LC-MS/MS (Fig. 4C). S121 phosphorylation in the H2A C-tail peptides containing unmodified K119 (blue in Fig. 4D) was detected only when Bub1C was added, indicating that the peptide may be a good substrate for Bub1C (Fig. 4D). On the other hand, the amount of S121 phosphorylation was much lower in the H2A C-tail peptides that contain malonylated K119 (red in Fig. 4D). To exclude that the difference of ionization efficiency in mass spectrometry may affect our analysis, we compared that ionization efficiency between the two phosphorylated peptides (VPNINAHLLPKTSphosGRTGK and VPNINAHLLPKmalTSphosGRTGK) and found that it was similar (see Methods). Therefore, we concluded that Hta1-K119 malonylation inhibits the phosphorylation of H2A-S121 by Bub1. This result is consistent with the results from the H2A-K119 malonyl-mimetic mutant protein experiments, suggesting that the phenotypes of H2A-K119 malonyl-mimetic mutants likely reflect the effects of malonylated K119.

### H2A-K119 malonylation inhibits interaction between Bub1 and H2A

**Figure 5:**
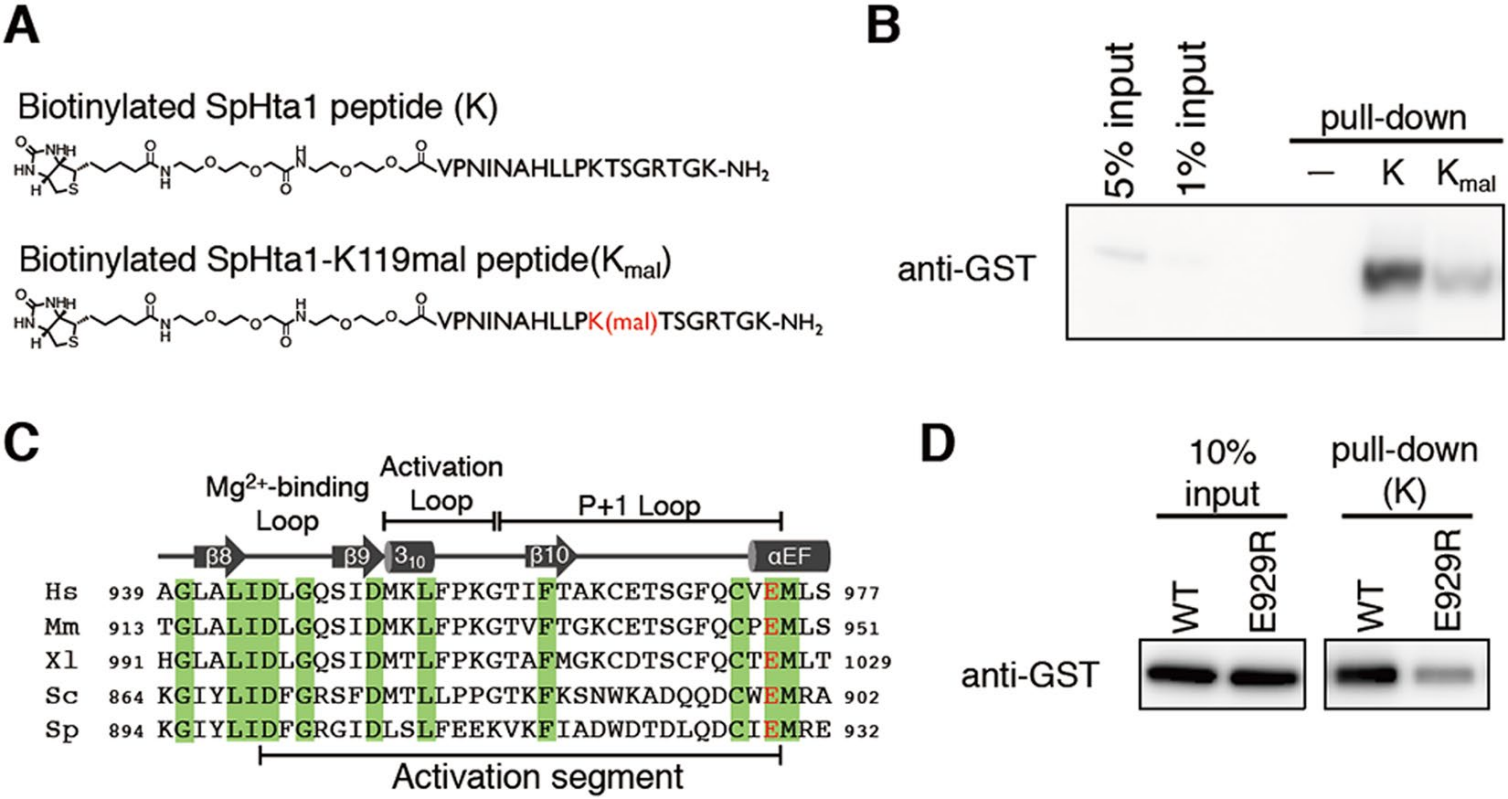
Bub1-H2A interaction through the kinase domain of Bub1 is affected by H2A-K119 malonylation. (**A**) The structures of unmodified or K119-malonylated SpHta1-C-tail peptides conjugated with biotin. Unmodified peptide is abbreviated as K, and K119-malonylated peptide is abbreviated as K_mal_ in later experiments. (**B**) Recombinant GST-SpBub1C protein was incubated with peptide K or K_mal_, and pulled down by streptavidin-coated beads. Input and bead-bound GST-spBub1C was detected by western blotting with anti-GST antibody. (**C**) The alignment of the amino acid sequences of the activation segment of Bub1. Identical residues are shaded green. Hs, *Homo sapiens*; Mm, *Mus musculus*; Xl, *Xenopus laevis*; Sc, *Saccharomyces cerevisiae*; Sp, *Schizosaccharomyces pombe*. The residue identical to E929 of fission yeast is in red. (**D**) Wild-type or E929R mutant recombinant protein of GST-SpBub1C were incubated with peptide K, and pulled down by streptavidin coated beads in the presence of 75 mM NaCl. Input and bead-bound GST-SpBub1C were detected by western blotting with anti-GST antibody. The grouping of images from different parts of the same gel is indicated by dividing lines. Uncropped images of (**B**) and (**D**) are shown in Fig. S6.

Finally, we addressed how H2A-K119 malonylation inhibits H2A-S121 phosphorylation by Bub1. We hypothesized that H2A-K119 malonylation might inhibit interaction between Bub1 and H2A. To investigate this hypothesis, we synthesized N-terminally biotinylated H2A-C-tail peptides (V109–K126 of SpHta1) with and without K119 malonylation (Fig. 5A), and examined the interactions between recombinant Bub1C proteins and these peptides. The K119-unmodified peptides efficiently pulled down Bub1C, whereas the K119-malonylated peptides did not (Fig. 5B). We also conducted a similar pull-down assay using budding yeast’s H2A-C-tail peptides (L109–K126 of ScHta1, Fig. S5A) and Bub1C protein and obtained similar results (Fig. S5B). These data indicate that H2A-K119 malonylation inhibits the interaction between Bub1 and H2A, which is likely the reason why H2A-K119 malonylation inhibits Bub1-dependent phosphorylation of H2A.

**Figure 6:**
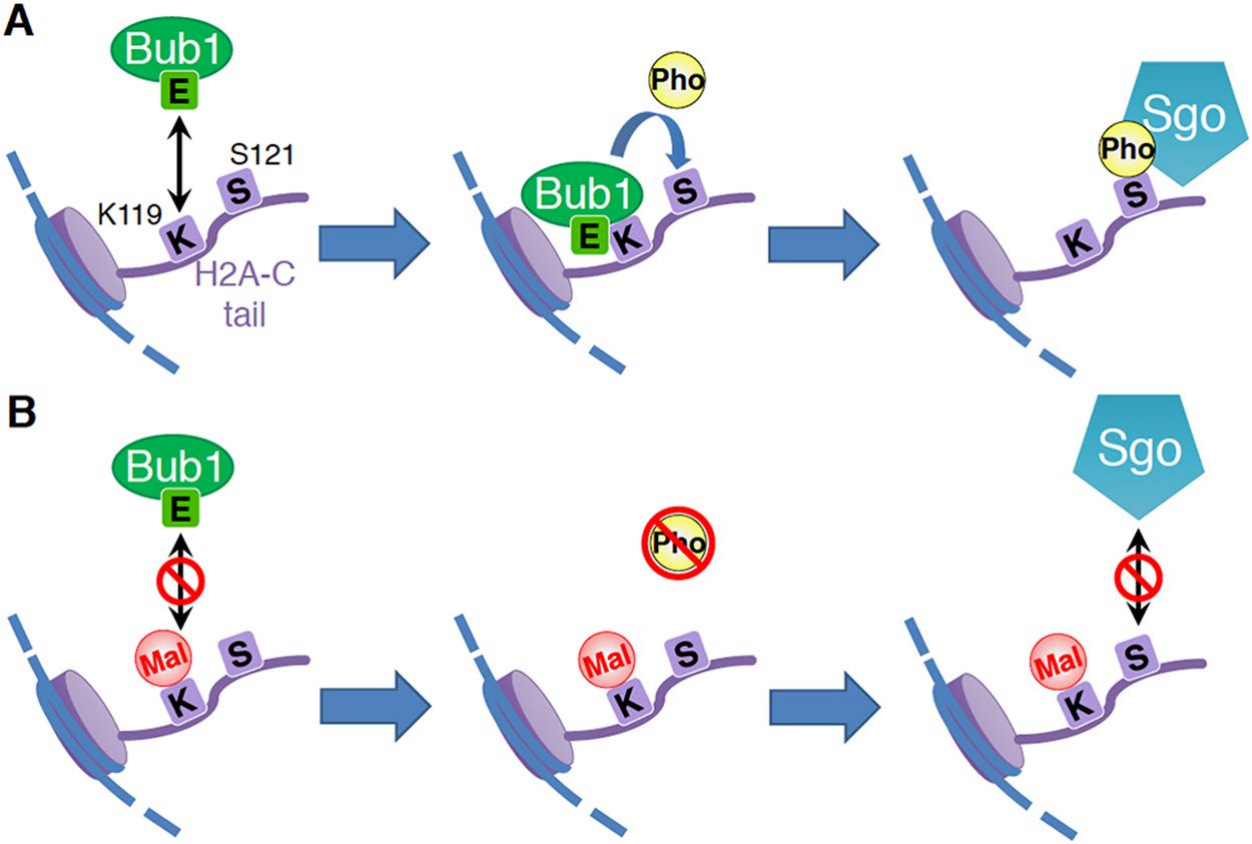
The model of H2A recognition by Bub1 and the effects of H2A-K119 malonylation. (**A**) When H2A-K119 is not modified, Bub1 recognizes the H2A-C tail, probably via electrostatic interaction between conserved glutamic acid (**E**) within Bub1’s kinase domain and K119 of H2A. Bub1 then catalyzes phosphorylation (Pho) at serine 121, which is read by shugoshin proteins (Sgo). (**B**) When H2A-K119 is malonylated (Mal), Bub1 fails to recognize H2A. The loss of H2A phosphorylation leads to delocalization of centromeric shugoshin and mis-segregation of chromosomes during mitosis and meiosis.

It has been reported that P + 1 loop within the kinase domain of human Bub1 is important for interaction with its substrate Cdc20^26^. On the other hand, it remains unclear how Bub1 recognizes H2A. As our data suggest that the cationic property of K119 is important for the interaction between Bub1 and H2A, we hypothesized that anionic amino acids near the P + 1 loop of Bub1 may be critical for recognizing H2A as a substrate via electrostatic interaction. We focused on the glutamic acid (E929 of fission yeast Bub1) that is located at the end of the P + 1 loop, as this glutamic acid is widely conserved among budding yeast, fission yeast, and other eukaryotes including human (Fig. 5C). Intriguingly, the E929R mutation in SpBub1 dramatically reduced interaction between Bub1 and H2A (Fig. 5D), suggesting that interaction between the P + 1 loop of Bub1, which contains the conserved glutamic acid, and H2A C-terminus tail, which contains K119, may be crucial for H2A recognition by Bub1 (Fig. 6).

## Discussion

For faithful chromosome segregation during mitosis and meiosis, Bub1 catalyzes phosphorylation of histone H2A at serine 121 in fission yeast and budding yeast (or threonine 120 in human) and recruits shugoshin proteins at the centromeres^21,27–29^. In this study, we report that malonylation of histone H2A at lysine 119 has an inhibitory role in the regulation of H2A phosphorylation. Although the biological significance of the mechanisms *in vivo* remains to be elucidated, it would be a potentially important regulation of chromosome segregation. A model of the functions of H2A-K119 malonylation is shown in Fig. 6. Bub1 recognizes H2A via electrostatic interaction between Bub1’s kinase domain, which contains the conserved glutamic acid, and H2A C-terminus tail, which contains K119, and catalyzes phosphorylation at serine 121 of H2A, which is read by shugoshin proteins. When H2A-K119 is malonylated, Bub1 fails to recognize H2A, leading to the loss of H2A phosphorylation and thus the delocalization of centromeric shugoshin and the subsequent mis-segregation of chromosomes during mitosis and meiosis. Analysis of the co-crystal structure of Bub1 and the H2A C-tail peptide may be informative in clarifying this model.

Our study demonstrates the first example of crosstalk between non-acetyl acylation and phosphorylation. Inhibition of H2A-S121 phosphorylation by H2A-K119 malonylation was observed when H2A-C tail peptides were used as substrates, so this cross-regulation occurs *in cis*. So far, a few examples of cross-regulation *in cis* between acetylation and phosphorylation have been reported. H3-S10 phosphorylation promotes acetylation of H3-K14 by Gcn5 *in vitro*^30^. This is likely mediated by the direct interaction of Gcn5 with the phosphorylated H3-S10 residue. In contrast, H3-S10 phosphorylation completely blocks acetylation of H3-K9^31^, probably by blocking the access of HATs to H3-K9. Acetylation of H2B-K11 inhibits phosphorylation of H2B-S10 by Ste20^32^. Our data suggest that not only acetylation, but also non-acetyl acylations at lysine residues, may influence the phosphorylation of nearby serine/threonine residues. The fact that various non-acetyl acylations have been identified at a number of lysine residues in histones indicates that the occurrence of cross-regulation is much more widespread than we have previously supposed. For example, various types of non-acetyl acylations (crotonylation, butyrylation, succinylation, 2-hydroxyisobutyrylation, and β-hydroxybutyrylation) have been detected at H3K27^4,11^, possibly inhibiting H3S28 phosphorylation. It is therefore important to address to what extent different types of acylation at lysine residues can affect nearby phosphorylation.

It is unknown whether H2A-K119 malonylation is catalyzed by a specific malonyltransferase or introduced by malonyl-CoA via a non-enzymatic mechanism. In either case, the level of H2A-K119 malonylation may fluctuate according to metabolic conditions, as intracellular concentrations of malonyl-CoA depend on the metabolic status of the cell^33,34^. Establishing methods to quantitatively measure the stoichiometry of H2A-K119 malonylation will be important for further understanding of our findings. Our results indicate that H2A-K119 malonylation must be removed for proper centromeric localization of shugoshin proteins and faithful chromosome segregation during mitosis and meiosis. As Sirt5 can remove lysine malonylation in human cells, a yeast sirtuin homolog, such as Sir2, might remove H2A-K119 malonylation in budding yeast or fission yeast. Both H2A-K119 and H2A-S121 are widely conserved between yeasts and higher eukaryotes including humans (Fig. 3A), suggesting that this novel crosstalk might be conserved as well in humans. In the congenital metabolic disease malonyl-CoA decarboxylase deficiency (MCD), malonyl-CoA fails to be broken down into acetyl-CoA and carbon dioxide, and the resulting accumulation of cellular malonyl-CoA may lead to excess histone malonylation. Therefore, investigating whether H2A-S121 phosphorylation and shugoshin localization are defective in MCD patients may be important to understanding the molecular mechanisms of such metabolic diseases.

## Methods

### Construction and analyses of budding yeast strains

All strains used in this study are listed in Table S1. To generate the series of point mutants of H2A and H2B, plasmids bearing a centromere sequence (CEN), a *HIS3* or *ADE2* marker, and gene cassettes of *HTA1 and HTB1* carrying each mutation were transformed into a host strain in which the endogenous H2A and H2B genes had been deleted, and a plasmid containing wild type *HTA1* and *HTB1* genes with an *URA3* marker had previously been introduced. First, transformants were selected by the introduced marker, and then counter-selected by 5-FOA to choose clones which had lost the originally introduced plasmid bearing wild type *HTA1 and HTB1*. To generate the series of point mutants of H3 or H4, we followed the same strategy, using plasmids with a centromere sequence, a *TRP1* marker, and *HHT2 and HHF2* gene cassettes carrying each mutation, and a host strain in which the endogenous H3 and H4 genes had been deleted and a plasmid containing wild type *HHT2* and *HHF2* genes with an *URA3* marker already introduced. Tagging of endogenous *SPC42*^+^ by mCherry or *SGO1*^+^ by GFP was performed using the PCR-generated DNA constructs as previously reported^22^. To measure the frequencies of non-disjunction of *CEN5*-GFP, budding yeast cells were cultured in YPD medium for 7 hours at 29 °C in the presence of 10 µg/mL of benomyl, fixed by methanol, and stained by DAPI and Calcofluor. To observe mitotic ScSgo1-GFP localization, budding yeast cells expressing Sgo1-GFP and Spc42-mCherry were grown in YE4S medium at 25 °C until the OD_590nm_ reached 0.15-0.3, fixed by methanol, and stained by DAPI.

### Construction and analyses of fission yeast strains

All strains used in this study are listed in Table S1. Every *h2a* mutant strain was generated as previously described^21^. Specifically, the genomic *hta1* and *hta2* DNA fragments carrying each mutation were amplified by PCR, and then transformed into *hta1::ura4*^+^ and *hta2::ura4*^+^ strains respectively. Individual single integrants were selected by 5-FOA and confirmed by PCR. After crossing both integrants of *hta1* and *hta2*, double mutants were selected. To visualize tubulin, *P*_*adh15*_ (a weak version of the *adh1*^+^ promoter)-*mCherry*-*atb2*^+^ was integrated at the Z locus using the *nat*^*r*^ marker. To measure the centromeric Sgo2-GFP signal during mitosis, fission yeast cells expressing Sgo2-GFP and mCherry-Atb2 were grown in YE4S medium until the OD_590nm_ reached 0.5, fixed by methanol, and stained by DAPI. To observe SpSgo1-GFP localization during meiosis I, homothallic fission yeast strains were grown in YE4S medium at 25 °C until the culture became saturated, spotted on SPA plates, and incubated at 25 °C for 11 hours. Zygotes were arrested at metaphase I by shutting down both *slp1*^+^ and *cut23*^+^ genes by the substitution of their promoter to that of the *rad21*^+^ gene. To monitor meiotic *cen2*-GFP segregation of fission yeast, haploid strains of opposite mating types with and without *cen2*-GFP were independently grown in YEA medium at 26.5 °C until the OD_590nm_ reached 0.2–0.3, spotted on SPA plates after being combined, and incubated at 26.5 °C for 12 hours.

### Immunoblotting

Cell extracts of budding yeast were prepared as previously described^35^. *S. cerevisiae* cells (3 mL in YPD, OD_600_ = 1) were harvested and pre-treated with 2 M LiAc. After cells were centrifuged, pellet was treated with NaOH for 5 min on ice. After centrifugation, cells were resuspended in 50 µL 2× sample buffer and boiled for 5 min. Cell extracts of fission yeast were prepared using glass beads as previously described^36^. Proteins were separated by SDS-PAGE using 5–20% or 5–10% Extra PAGE One Precast Gels (Nacalai), and transferred to Immobilon P PVDF membranes (Millipore). For blocking, 5% skim milk (Nacalai) in TBS containing 0.1% Tween-20 (Santa Cruz) was used. The following antibodies were used for western blot analysis. Primary antibodies: H2A (active motif, 39945, 1:1,000); GFP (Takara, 632592, 1:1,000); TAT1 (a gift from K. Gull)^37^. Secondary antibodies: anti-rabbit IgG-HRP (GE Healthcare, NA934V, 1:10,000); anti-mouse IgG-HRP (GE Healthcare, NA931V, 1:10,000). Chemiluminescence generated by Luminata Forte HRP substrate (Millipore) was detected by LAS 4000 mini (GE Healthcare).

### Chromatin immunoprecipitation (ChIP) assay

Logarithmically growing cells (OD = 0.25) at 29 °C were fixed by 11% formaldehyde solution (11% formaldehyde, 100 mM NaCl, 1 mM EDTA (pH 8.0), 0.5 mM EGTA, 50 mM Tris-HCl (pH 7.5)) for 10 min at 29 °C and for >50 min at 4 °C. After washing by Buffer1 (50 mM HEPES (pH 7.5), 140 mM, NaCl 1 mM, EDTA (pH 8.0), 1% Triton X-100, 0.1% Na Deoxycholate) four times, pellets were freezed with liquid nitrogen and store at −80 °C. After cells were broken by glass beads with Buffer1 containing 5× cOmplete (Sigma-Aldrich), PhosSTOP (Sigma-Aldrich), and 1 mM PMSF, and sonicated by Ultrasonic Disruptor (TOMY Digital Biology), the supernatants were collected as whole cell extract (WCE). After 150 µL of WCE (protein concentration: 1.2 mg/mL) was incubated with anti-H2A (active motif, 39945) or normal rabbit IgG (Santa Cruz Biotechnology, sc-2027) for 30 min on ice, 10 µL of Dynabeads Protein G (Veritas) was added to WCE-antibody mixture and incubated for 2 hours at 4 °C. After washing by 0.5 mL Buffer1, 0.5 mL Buffer1’ (50 mM HEPES (pH 7.5), 500 mM, NaCl, 1 mM, EDTA (pH 8.0), 1% Triton X-100, 0.1% Na Deoxycholate), 0.5 mL Buffer 2 (40 mM Tris-HCl (pH 8.0), 250 mM, LiCl, 1 mM, 0.5% NP-40, 0.5% Na Deoxycholate), and 0.5 mL TE, the co-immunoprecipitated DNA was extracted at 65 °C for 15 min with Buffer containing 20 mM Tris-HCl, 100 mM NaCl, 20 mM EDTA, 0.1% SDS. The supernatant was collected and incubated at 75 °C for overnight, after which 3 μL Proteinase K (Takara, 9034) was added and the incubation was continued for an additional 3 hours at 55 °C. DNA was purified using a PCR Clean-Up Mini Kit (FAVORGEN) before PCR. DNA prepared from whole cell extracts or immunoprecipitated fractions was analyzed by quantitative PCR with the LightCycler 480 system (Roche) using SYBR Green I Master (Roche). The amount of co-immunoprecipitated DNA was divided by that of total DNA in WCE to calculate H2A IP (%) or IgG IP (%). Then H2A IP (%) was subtracted by IgG IP (%) to calculate IP (%), which corresponds the Y-axis of the graph for ChIP experiments.

### Construction of plasmids

To express GST-ScBub1C and GST-SpBub1C, the C-terminus of budding yeast *BUB1*^+^ ORF (631 to 1021 in the amino acid sequence) or fission yeast *bub1*^+^ ORF (633 to 1041 in the amino acid sequence) was amplified by PCR and cloned into the pGEX6p-2 vector. To express GST-ScHta1 and GST-SpHta1, the full-length of either budding yeast *HTA1*^+^ ORF or fission yeast *hta1*^+^ ORF were cloned as described above. All of the point mutations of *bub1* and histone genes were introduced by PCR mutagenesis with PrimeSTAR Max DNA polymerase (Takara).

### Purification of recombinant proteins

The recombinant proteins of GST-SpBub1C, GST-ScBub1C, GST-SpHta1, and GST-ScHta1 were produced in *E*. *coli* (BL21C^+^) using the pGEX6p-2 vector. All of the GST-fused proteins were affinity-purified by glutathione sepharose 4B resin (GE Healthcare) and eluted by adding glutathione. GST-SpBub1C, GST-Bub1C (E929R), and GST-ScBub1C were further purified by the gel-filtration system AKTA pure M1 with Superdex 200 10/300 GL column (GE Healthcare).

### *In vitro* phosphorylation assay with recombinant Hta1 proteins

The mixture of recombinant kinase GST-Bub1C and substrate GST-Hta1 (wild type or mutants) were incubated in kinase buffer (50 mM Tris-HCl (pH 7.5), 10 mM MgCl_2_, 1% Triton X-100) for 30 min at 30 °C in the presence of [γ-^32^P] ATP. Reacted proteins were separated by SDS-PAGE. Loading amount of GST-Hta1 proteins was checked by CBB staining and the phosphorylation was detected by autoradiography.

### *In vitro* phosphorylation assay with fission yeast’s Hta1-C-tail peptides

A 1 mM mixture of unmodified and K119-malonylated SpHta1-C-tail peptides was incubated in kinase buffer containing 100 µM ATP and 1 mg/ml BSA with or without recombinant GST-SpBub1C for 30 min at 30 °C. After the reaction, GST-SpBub1C was immediately denatured and precipitated by adding 4% trichloroacetic acid. After centrifugation, the supernatant was analyzed by LC–MS/MS in a 50-fold dilution with Milli Q.

### Peptide pull down assay

First, unmodified or K119-malonylated Hta1-C-tail peptides conjugated with biotin were captured by Dynabeads M-280 streptavidin (invitrogen). After washing by PBS three times, the peptide-bound beads were mixed with recombinant GST-Bub1C protein in buffer-P (20 mM Tris-HCl (pH 7.5), 10 mM MgCl_2_, 1% Triton X-100, 1 mg/ml BSA) and incubated at 4 °C for 1 hour. After washing with buffer-P without BSA twice, the bead-bound proteins were analyzed by immunoblotting with an anti-GST antibody (wako, 013-21851).

### LC–MS/MS analyses

LC–MS/MS analyses were conducted using an AB Sciex Triple TOF 4600 equipped with an Eksigent ekspert microLC 200. LC was carried out as follows: 3C18-CL-120 column (0.5 mm l.D × 100 mm) using a linear gradient of 2–35% acetonitrile with 0.1% formic acid (*v*/*v*) versus water with 0.1% formic acid (*v*/*v*) over 6 min at 40 °C with a flow rate of 20 µL min^−1^ after 1 min equilibration. The amount of the injected samples was 5 µL. The eluent was monitored by on-line quadrupole time-of-flight mass spectrometer MS, operated in positive ion mode. Data-dependent analyses were conducted for characterization of synthetic peptides. Data-independent analyses were conducted for analysis of *in-vitro* phospholylation, with targeted precursor ions and collision energies of 496.27 (CE = 25) for the S121phos peptide and 517.27 (CE = 25) for the S121phos/K119mal peptide. Data analysis was carried out using PeakView software (AB Sciex, version 1.2.0.3). We compared ionization efficiency between the two phosphorylated peptides (VPNINAHLLPKTSphosGRTGK and VPNINAHLLPKmalTSphosGRTGK) as follows: The two phosphorylated peptides were mixed with BSA (0.125 mg/mL) and unphospholylated peptides (VPNINAHLLPKTSGRTGK and VPNINAHLLPKmalTSGRTGK, 2 mM each) in buffer (20 mM Tris pH 7.5, 4 mM MgCl_2_, 1% Triton). After incubation at 30 degree for 30 min, TCA was added to the final concentration of 4%, and incubated on ice for 30 min. After centrifugation (14000 rpm, 10 min), supernatant was analyzed by LC-MS/MS. Peak area was calculated on the software, and the obtained ratio of the peak area of unmalonylated one to malonylated one was 1.17 ± 0.05 (N = 5). Therefore, we considered that the difference of ionization efficiency did not affect our conclusion by LC-MS-based experiments.

### Data Availability

The datasets generated and/or analyzed during the current study are available from the corresponding author on reasonable request.

## Electronic supplementary material

Supplementary info
