# Supplementary material for: Malonylation of histone H2A at lysine 119 inhibits Bub1-dependent H2A phosphorylation and chromosomal localization of shugoshin proteins
Source: Sci Rep. 2018 May 16;8:7671. doi: 10.1038/s41598-018-26114-z (PMC5956101; doi:10.1038/s41598-018-26114-z)
Supplement: Supplementary file 1 — Supplementary info [file 41598_2018_26114_MOESM1_ESM.pdf]

Supplementary Information for:

Malonylation of histone H2A at lysine 119 inhibits Bub1-dependent H2A phosphorylation and chromosomal localization of shugoshin proteins.

Tadashi Ishiguro[1,2], Kana Tanabe[1,2], Yuki Kobayashi[1,2], Shinsuke Mizumoto[1], Motomu Kanai[1,2], Shigehiro A. Kawashima[1,2,*]

[1]Graduate School of Pharmaceutical Sciences, The University of Tokyo, 7-3-1 Hongo, Bunkyo-ku, Tokyo 113-0033, Japan, [2] JST-ERATO, Kanai Life Science Catalysis Project, 7-3-1, Hongo, Bunkyo-ku, Tokyo 113-0033, Japan

*Corresponding Author:
Shigehiro A. Kawashima
Email: skawashima@mol.f.u-tokyo.ac.jp

Contents:

1. Synthesis of peptides
2. Supplementary Figures

1. Synthesis of peptides

Reagents

NovaPEG-Rink Amide resin were purchased from Novabiochem. 2-Chlorotrityl Chloride Resin was purchased from Peptides International (Kentucky, USA). Fmoc-amino acids, *O*-(7-Aza-1H-benzotriazol-1-yl)-*N*,*N*,*N'*,*N'*-tetramethyluronium hexafluorophosphate (HATU), and *O*-(6-Chloro-1H-benzotriazol-1-yl)-*N*,*N*,*N'*,*N'*-tetramethyluronium hexafluorophosphate (HCTU) were purchased from Peptides Institute, Inc. (Osaka, Japan). 1-hydroxybenzotriazole (HOBt) and Fmoc-Ser{PO(OBzl)OH}-OH were purchased from Watanabe Chemical (Hiroshima, Japan). (+)-Biotin and dichloromethane was purchased from Wako Pure Chemical Industries (Osaka, Japan). *N*,*N*-Dimethylformamide (DMF), acetonitrile, and dry solvents of tetrahydrofuran (THF) were purchased from Kanto Chemical (Tokyo, Japan). 9-(Fmoc-amino)-4,7-dioxanonanoic acid, mono-*tert*-butyl malonate, *N*,*N'*-Diisopropylcarbodiimide (DIC) and trifluoroacetic acid (TFA) were purchased from Tokyo Chemical Industry (Tokyo, Japan). Triisopropylsilane (TIPS) was purchased from Sigma-Aldrich Japan (Tokyo, Japan).

Analytical HPLC for fission yeast's Hta1-C-tail peptides

Analytical HPLC was conducted by using a Shimadzu HPLC system equipped with an SPD-20A spectrometer, LC-20AD pumps, and a DGU-20A3R degasser with a linear gradient of 0–100% acetonitrile in 0.1% aqueous TFA over 40 min after 2 min equilibration with a flow rate of 0.9 mL/min, using a YMC-Triart C18 column (4.6 mm I.D. x 150 mm).

Preparative HPLC for fission yeast's Hta1-C-tail peptides

Preparative HPLC was conducted by using a Shimadzu HPLC system equipped with an SPD-20A spectrometer, and LC-6AD pumps, with a linear gradient of 0–100% acetonitrile in 0.1% aqueous TFA over 100 min after 5 min equilibration with a flow rate of 3 mL/min using a YMC-Triart C18 column (10 mm I.D. x 250 mm).

Syntheses of fission yeast's Hta1-C-tail peptides

All the peptides were synthesized on a solid phase using NovaPEG-Rink Amide resin. Fmoc-amino acids (4 eq.) and mono-tert-butyl malonate (4 eq.) were introduced using an HATU–iPr_2NEt method or an HOBt–DIC method in DMF (4 eq. each) for 40 min (for Fmoc-amino acids) at room temperature after removal of each Fmoc group with 20% piperidine-DMF for 10 min. The peptides were cleaved from the resin by treatment with TFA in the presence of TIPS and H_2O (95:2.5:2.5) for 60 min at room temperature, concentrated under reduced pressure, and precipitated with ether to afford crude peptides, which were purified with preparative HPLC to afford product peptides as white solids after lyophilization. Fmoc-Lys(tert-butyl malonate)-OH was used for the introduction of malonylated lysine, and Fmoc-Ser{PO(OBzl)OH}-OH was used for the introduction of phosphorylated serine. 9-(Fmoc-amino)-4,7-dioxanonanoic acid was used for the introduction of TEG moiety.

VPNINAHLLPKTSGRTGK. ESI–MS *m/z* Calcd: 634.71 $[M+3H]^{3+}$, Found: 634.71; Retention Time: 13.9 min; Purity: >95%.

VPNINAHLLPKTSphosGRTGK. ESI–MS *m/z* Calcd: 661.36 $[M+3H]^{3+}$, Found: 661.36; Retention Time: 14.1 min; Purity: >95%.

VPNINAHLLPKmalTSGRTGK. ESI–MS *m/z* Calcd: 497.79 $[M+3H]^{3+}$, Found: 497.78; Retention Time: 14.6 min; Purity: >95%.

VPNINAHLLPKmalTSphosGRTGK. ESI–MS *m/z* Calcd: 517.78 $[M+3H]^{3+}$, Found: 517.78; Retention Time: 14.9 min; Purity: >95%.

Biotin-(TEG)-(TEG)-VPNINAHLLPKTSGRTGK. ESI–MS *m/z* Calcd: 605.34 $[M+4H]^{4+}$, Found: 605.34; Retention Time: 16.7 min; Purity: >95%.

Biotin-(TEG)-(TEG)-VPNINAHLLPKmalTSGRTGK. ESI–MS *m/z* Calcd: 626.84 $[M+4H]^{4+}$, Found: 626.84; Retention Time: 17.4 min; Purity: >95%.

Analytical HPLC for budding yeast's Hta1-C-tail peptides

Analytical HPLC was conducted by using a JASCO HPLC system equipped with an UV-2075Plus UV detector, PU-2080Plus pumps, CO-2065Plus column oven, MX-2080-32 mixer and a DG-2080-54 degasser with a linear gradient of 0–100%

acetonitrile in 0.1% aqueous TFA over 40 min after 2 min equilibration with a flow rate of 0.9 mL/min, 40 ºC, using a YMC-Triart C18 column (4.6 mm I.D. x 150 mm).

Preparative HPLC for budding yeast's Hta1-C-tail peptides

Preparative HPLC was conducted by using a JASCO HPLC system equipped with an UV-2075Plus UV detector, PU-2086Plus pumps, CO-2065Plus column oven, MX-2080-32 mixer and a DG-2080-54 degasser with a following 0.1% aqueous TFA /acetonitrile gradient conditions; 0–5 min, 0% acetonitrile, 5–25 min, 0 to 30% acetonitrile, 25–55 min, 30 to 60% acetonitrile, flow rate of 10 mL/min, 40 ºC using a YMC-Triart C18 column (30 mm I.D. x 250 mm).

Syntheses of budding yeast's Hta1-C-tail peptides

All the peptides were synthesized on a solid phase using NovaPEG-Rink Amide resin. Fmoc-amino acids (2.5 eq.) and mono-tert-butyl malonate (2.5 eq.) were introduced using an HCTU–iPr_2NEt method in DMF (2.5 eq. each) for 0.5–20 hours (for Fmoc-amino acids) at room temperature after removal of each Fmoc group with 20% piperidine-DMF for 10–60 min. The peptides were cleaved from the resin by treatment with TFA in the presence of TIPS and H_2O (95:2.5:2.5) for 60 min at room temperature, concentrated under reduced pressure, and precipitated with ether to afford crude peptides, which were purified with preparative HPLC to afford product peptides as white solids after lyophilization. Malonylation of Lys was conducted by synthetic method as below. After coupling of Fmoc-Lys(4-methyltrityl)-OH by solid phase peptide synthesis, the 4-methyltrityl group of protected peptide-resin was deprotected with a solution of TFA/TIPS/dichloromethane (1:5:94). Then the deprotected peptide-resin was coupled with mono-tert-butyl malonate using HCTU-iPr_2NEt method in DMF (2.5 eq. each). Next coupling steps were conducted as described above. 9-(Fmoc-amino)-4,7-dioxanonanoic acid was used for the introduction of TEG moiety. MALDI-TOF-MS was obtained with a Shimadzu Biotech Axima ToF^2 spectrometer.

Biotinyl-(TEG)-(TEG)-LPNIHQNLLPKKSAKATK. MALDI-TOF-MS m/z Calcd: 2516.44 $[M+H]^+$, Found: 2516.53; Retention Time: 16.8 min; Purity: >95%.

Biotinyl-(TEG)-(TEG)-LPNIHQNLLPKmalKSAKATK. MALDI-TOF-MS m/z Calcd: 2557.44 $[M–CO_2]^+$, Found: 2557.52; Retention Time: 17.8 min; Purity: 93%.

2. Supplementary Figures

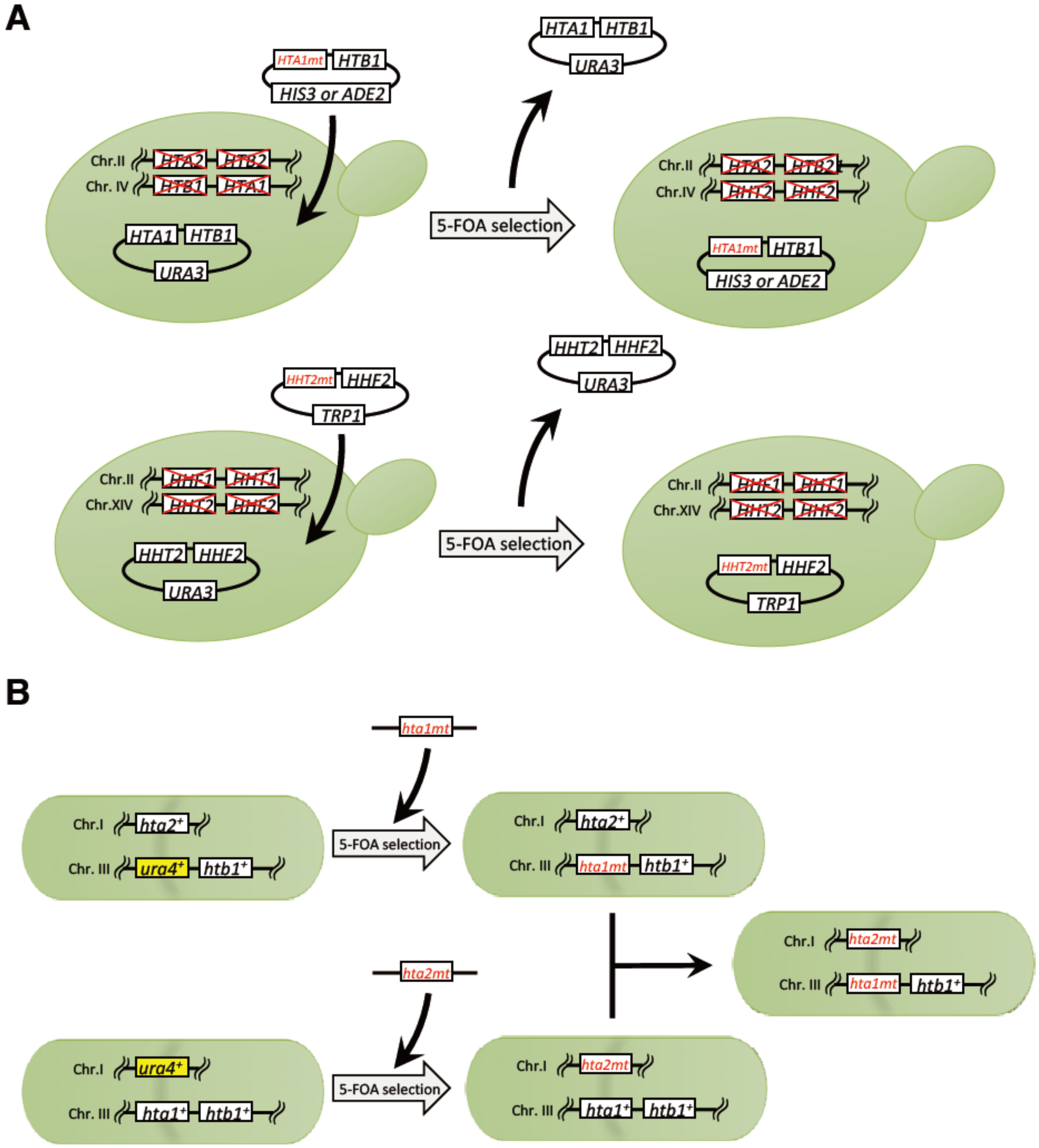

Figure S1. Methods to generate histone mutants of budding and fission yeast in this study. (A) Schematics of our strategy to generate a H2A/H2B mutant (upper panel), or a H3/H4 mutant (lower panel) in budding yeast. (B) Schematics of our strategy to generate a H2A mutant in fission yeast.

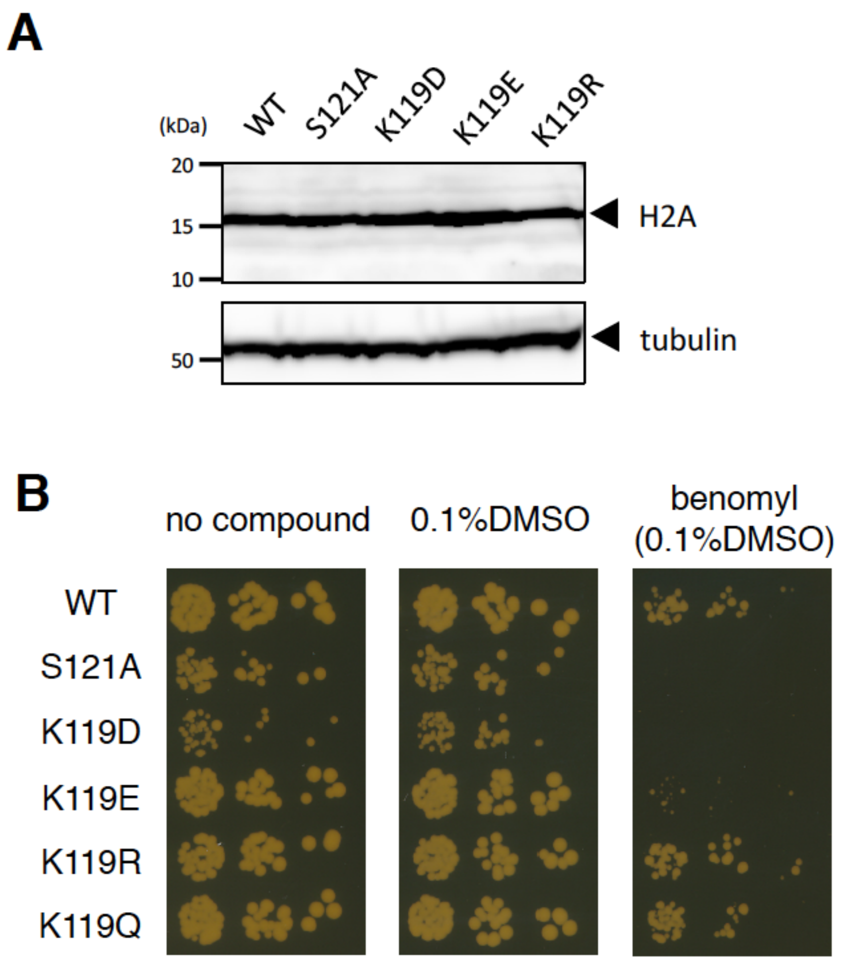

Figure S2. Analyses of budding yeast H2A-K119 malonyl-mimetic mutants. (A) The level of histone H2A in the indicated H2A mutant strains was estimated by western blotting with anti-H2A antibody. Tubulin is the loading control. (B) Serial dilutions (5000, 1000, 200, 40 cells) of WT and indicated H2A mutant strains of buddung yeast are grown on YPD plate, or YPD plate containing 0.1 % DMSO or benomyl (15 μg/ml, 0.1% DMSO), and incubated at 29℃ for 2 days.

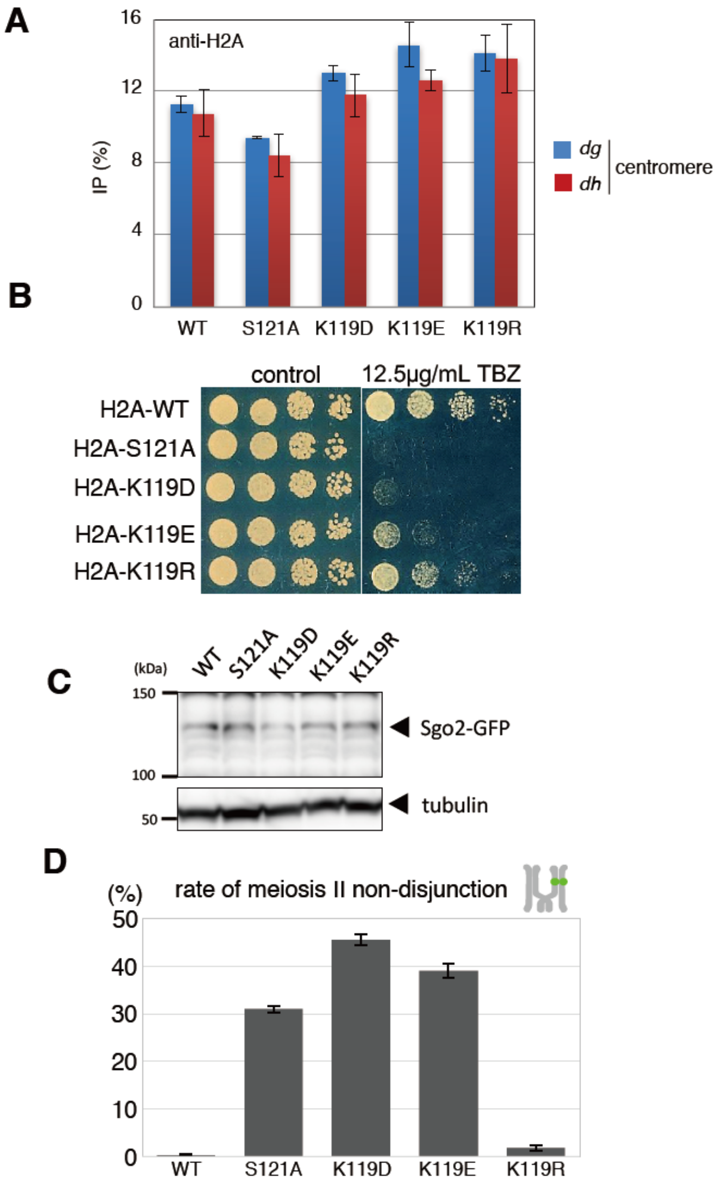

Figure S3. Analyses of fission yeast H2A-K119 malonyl-mimetic mutants. (A) CHIP analyses of histone H2A at centromeres (dg, dh). The graph shows the percentage of co-immunoprecipitated DNA with anti-H2A antibody per total DNA in whole cell extracts (error bar = range of 2 measurements). (B)Serial dilutions (5000, 1000, 200, 40 cells) of WT and indicated H2A mutant strains of fission yeast are grown on non-selective YE plate or YE plate containing TBZ (12.5 µg/ml), and incubated at 29℃ for 2 days. (C) The level of Sgo2-GFP in the indicated H2A mutant strains was estimated by western blotting with anti-GFP antibody. Tubulin is the loading control. (D) Sister chromatid segregation during meiosis was monitored by heterozygously labeled centromere of chromosome 2 with GFP (cen2-GFP). The graph shows the rate of non-disjunction at meiosis II. (n > 200, error bar = range of 2 experiments)

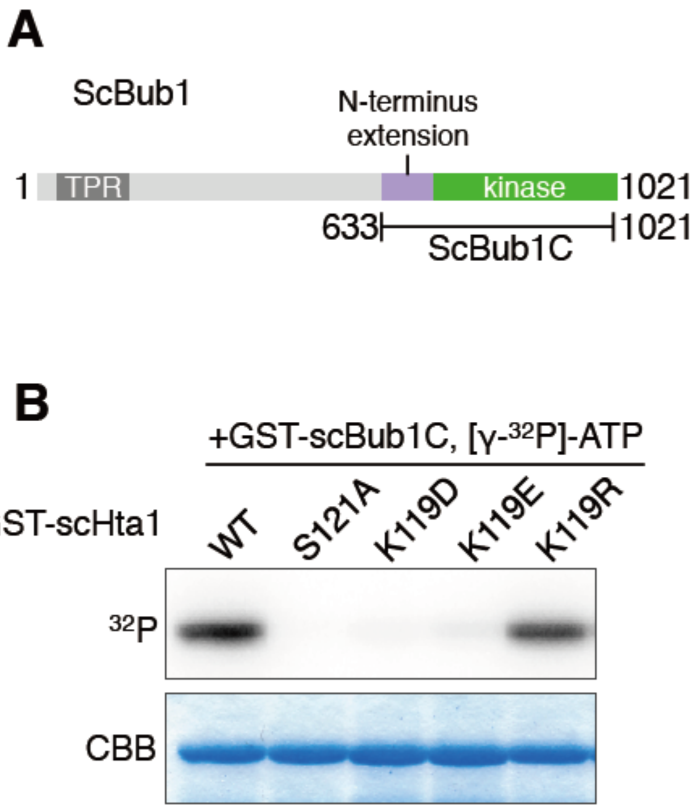

Figure S4. **The effects of H2A-K119 malonyl-mimetic mutation was conserved in budding yeast.** (A) Schematic of budding yeast's Bub1 (ScBub1). TPR, tetratricopeptide repeat; kinase, kinase domain. The C-terminus fragment of ScBub1 (ScBub1C) was used in (B). (B) The recombinant GST-ScHta1 proteins of wild-type (WT) or the indicated mutants were incubated with the recombinant GST-ScBub1C in the presence of [γ-^{32}P]-ATP. Incorporated ^{32}P was detected by autoradiography and protein loading amount was confirmed by CBB staining.

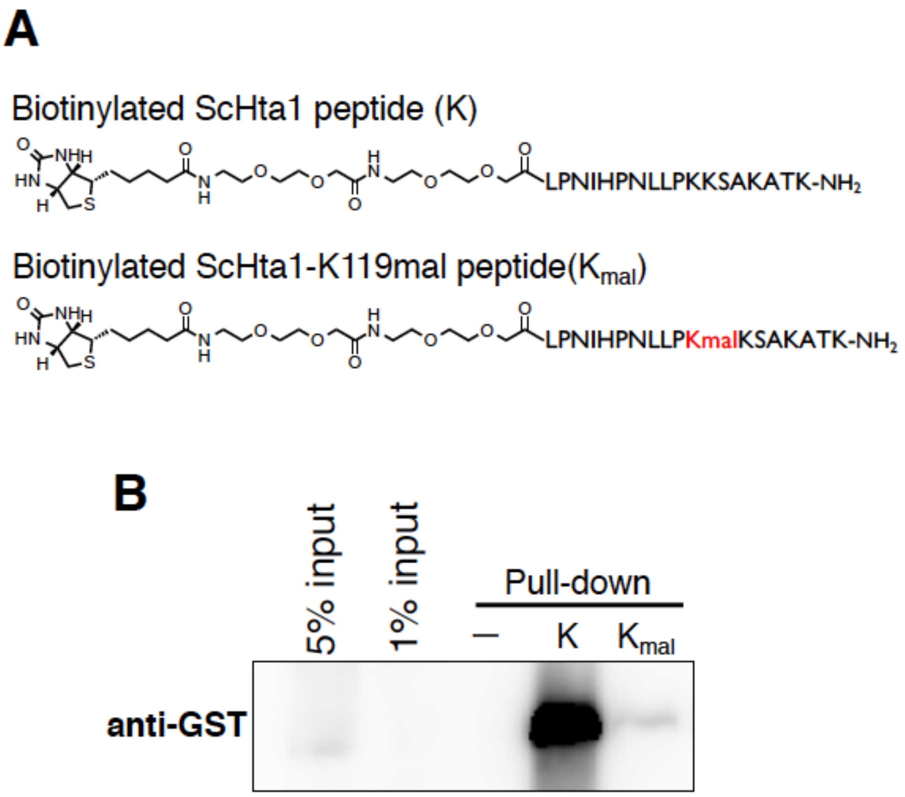

Figure S5. The effects of H2A-K119 malonylation was conserved in budding yeast. (A) Chemical structures of unmoodified or K119-malonylated ScHta1-C-tail peptides conjugated with biotin. The unmodified peptide is abbreviated as K, and K119-malonylated peptide is abbreviated as K_{mal} in (B). (B) The recombinant GST-ScBub1C protein was incubated with the unmodified or a K119-malonylated ScHta1-C-tail peptide, and pulled down by the streptavidin coated beads. GST-ScBub1C in the input and the collected beads was detected by western blotting with the anti-GST antibody.

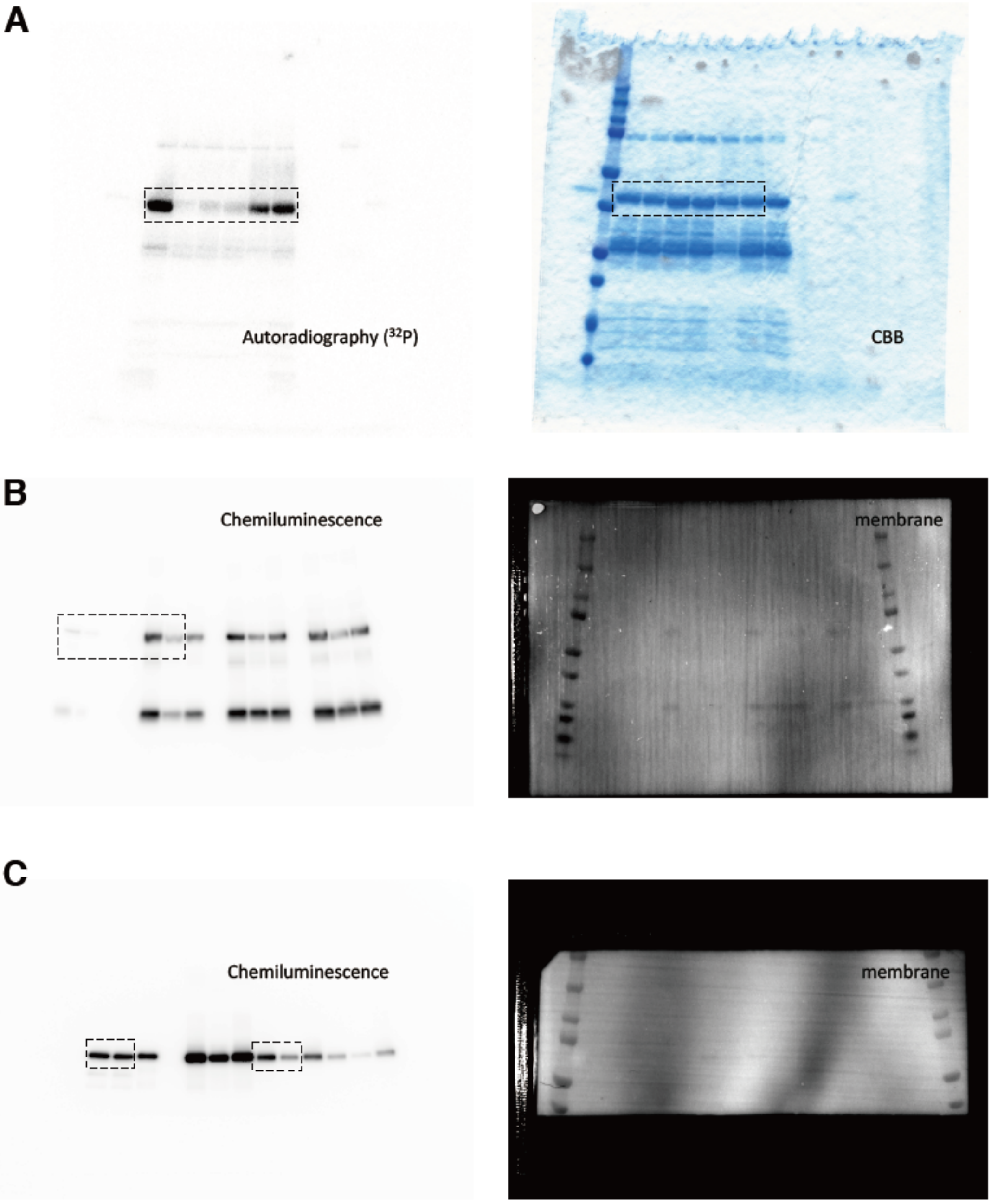

Figure S6. Uncropped images for Figures 4B, 5B, and 5D. (A) The whole gel image for Figure 4B. (B) The whole membrane for Figure 5B. (C) The whole membrane for Figure 5D. The left 2 lanes indicate input samples, and the right 2 lanes indicate pull down samples.

Table S1. Yeast strain list (sc: budding yeast, others: fission yeast)

Figure No.	strain name	genotype
Figure 1C	sc213	MATa (hta1-htb1)⊿::LEU2+ (hta2-htb2)⊿::TRP1 his⊿200 trp1⊿63 lys2-128δ ura3-52 leu2⊿1 pJH55(HTA1WT-HTB1WT HIS3 CEN)
	sc347	MATa (hta1-htb1)⊿::LEU2+ (hta2-htb2)⊿::TRP1 his⊿200 trp1⊿63 lys2-128δ ura3-52 leu2⊿1 pJH55(HTA1K13E-HTB1WT HIS3 CEN)
	sc348	MATa (hta1-htb1)⊿::LEU2+ (hta2-htb2)⊿::TRP1 his⊿200 trp1⊿63 lys2-128δ ura3-52 leu2⊿1 pJH55(HTA1K21E-HTB1WT HIS3 CEN)
	sc349	MATa (hta1-htb1)⊿::LEU2+ (hta2-htb2)⊿::TRP1 his⊿200 trp1⊿63 lys2-128δ ura3-52 leu2⊿1 pJH55(HTA1K119E-HTB1WT HIS3 CEN)
	sc350	MATa (hta1-htb1)⊿::LEU2+ (hta2-htb2)⊿::TRP1 his⊿200 trp1⊿63 lys2-128δ ura3-52 leu2⊿1 pJH55(HTA1WT-HTB1K37E HIS3 CEN)
	sc351	MATa (hta1-htb1)⊿::LEU2+ (hta2-htb2)⊿::TRP1 his⊿200 trp1⊿63 lys2-128δ ura3-52 leu2⊿1 pJH55(HTA1WT-HTB1K49E HIS3 CEN)
	sc291	MATa ade2-1 ura3-1 his3-11 trp1-1 leu2-3,112 can1-100 hht1Δhhf1Δ::LEU2 hht2Δhhf2Δ::HIS3 pRS414(HHT2WT-HHF2WT TRP1)
	sc326	MATa ade2-1 ura3-1 his3-11 trp1-1 leu2-3,112 can1-100 hht1Δhhf1Δ::LEU2 hht2Δhhf2Δ::HIS3 pRS414(HHT2WT-HHF2K31E TRP1)
	sc325	MATa ade2-1 ura3-1 his3-11 trp1-1 leu2-3,112 can1-100 hht1Δhhf1Δ::LEU2 hht2Δhhf2Δ::HIS3 pRS414(HHT2K79E-HHF2WT TRP1)
	sc298	MATa ade2-1 ura3-1 his3-11 trp1-1 leu2-3,112 can1-100 hht1Δhhf1Δ::LEU2 hht2Δhhf2Δ::HIS3 pRS414(HHT2WT-HHF2K77E TRP1)

Figure 2A	sc335	MATalpha ade2-1 ura3-1 hta1-htb1::CgTRP1 hta2-htb2::hphMX4 CEN5::tetO2x112::HIS3 leu2::TetR-GFP::LEU2 bar1 RAD5 [pRS412 HTA1-HTB1(ADE2)]
	sc341	MATalpha ade2-1 ura3-1 hta1-htb1::CgTRP1 hta2-htb2::hphMX4 CEN5::tetO2x112::HIS3 leu2::TetR-GFP::LEU2 bar1 RAD5 [pRS412 HTA1S121A-HTB1(ADE2)]
	sc338	MATalpha ade2-1 ura3-1 hta1-htb1::CgTRP1 hta2-htb2::hphMX4 CEN5::tetO2x112::HIS3 leu2::TetR-GFP::LEU2 bar1 RAD5 [pRS412 HTA1K119D-HTB1(ADE2)]
	sc339	MATalpha ade2-1 ura3-1 hta1-htb1::CgTRP1 hta2-htb2::hphMX4 CEN5::tetO2x112::HIS3 leu2::TetR-GFP::LEU2 bar1 RAD5 [pRS412 HTA1K119E-HTB1(ADE2)]
	sc336	MATalpha ade2-1 ura3-1 hta1-htb1::CgTRP1 hta2-htb2::hphMX4 CEN5::tetO2x112::HIS3 leu2::TetR-GFP::LEU2 bar1 RAD5 [pRS412 HTA1K119R-HTB1(ADE2)]
	sc340	MATalpha ade2-1 ura3-1 hta1-htb1::CgTRP1 hta2-htb2::hphMX4 CEN5::tetO2x112::HIS3 leu2::TetR-GFP::LEU2 bar1 RAD5 [pRS412 HTA1K119Q-HTB1(ADE2)]
Figure 2B	sc335	MATalpha ade2-1 ura3-1 hta1-htb1::CgTRP1 hta2-htb2::hphMX4 CEN5::tetO2x112::HIS3 leu2::TetR-GFP::LEU2 bar1 RAD5 [pRS412 HTA1-HTB1(ADE2)]
	sc341	MATalpha ade2-1 ura3-1 hta1-htb1::CgTRP1 hta2-htb2::hphMX4 CEN5::tetO2x112::HIS3 leu2::TetR-GFP::LEU2 bar1 RAD5 [pRS412 HTA1S121A-HTB1(ADE2)]
	sc338	MATalpha ade2-1 ura3-1 hta1-htb1::CgTRP1 hta2-htb2::hphMX4 CEN5::tetO2x112::HIS3 leu2::TetR-GFP::LEU2 bar1 RAD5 [pRS412 HTA1K119D-HTB1(ADE2)]
	sc339	MATalpha ade2-1 ura3-1 hta1-htb1::CgTRP1 hta2-htb2::hphMX4 CEN5::tetO2x112::HIS3 leu2::TetR-GFP::LEU2 bar1 RAD5 [pRS412 HTA1K119E-HTB1(ADE2)]
	sc336	MATalpha ade2-1 ura3-1 hta1-htb1::CgTRP1 hta2-htb2::hphMX4 CEN5::tetO2x112::HIS3 leu2::TetR-GFP::LEU2 bar1 RAD5 [pRS412 HTA1K119R-HTB1(ADE2)]
Figure 2C	sc221	MATa (hta1-htb1)⊿::LEU2+ (hta2-htb2)⊿::TRP1 his⊿200 trp1⊿63 lys2-128δ ura3-52 leu2⊿1

		SGO1-GFP<<kanr SPC42-mCherry<<natr pJH55(HTA1WT-HTB1WT HIS3 CEN)
	sc222	MATa (hta1-htb1)⊿::LEU2+ (hta2-htb2)⊿::TRP1 his⊿200 trp1⊿63 lys2-128δ ura3-52 leu2⊿1 SGO1-GFP<<kanr SPC42-mCherry<<natr pJH55(HTA1S121A-HTB1WT HIS3 CEN)
	sc401	MATa (hta1-htb1)⊿::LEU2+ (hta2-htb2)⊿::TRP1 his⊿200 trp1⊿63 lys2-128δ ura3-52 leu2⊿1 SGO1-GFP<<kanr SPC42-mCherry<<natr pJH55(HTA1K119D-HTB1WT HIS3 CEN)
	sc403	MATa (hta1-htb1)⊿::LEU2+ (hta2-htb2)⊿::TRP1 his⊿200 trp1⊿63 lys2-128δ ura3-52 leu2⊿1 SGO1-GFP<<kanr SPC42-mCherry<<natr pJH55(HTA1K119E-HTB1WT HIS3 CEN)
	sc405	MATa (hta1-htb1)⊿::LEU2+ (hta2-htb2)⊿::TRP1 his⊿200 trp1⊿63 lys2-128δ ura3-52 leu2⊿1 SGO1-GFP<<kanr SPC42-mCherry<<natr pJH55(HTA1K119R-HTB1WT HIS3 CEN)
Figure 3C, D	SAK4109	h- sgo2+-GFP<<kanr z::Padh15-mCherry-atb2+<<natr
	SAK4111	h- ade6 leu1 sgo2+-GFP<<kanr hta1-S121A hta2-S121A z::Padh15-mCherry-atb2+<<natr
	SAK4113	h- ade6-M210 leu1 ura4-D18 sgo2+-GFP<<kanr hta1-119KD hta2-119KD z::Padh15-mCherry-atb2+<<natr
	SAK4115	h+ ade6-M216 leu1 ura4-D18? sgo2+-GFP<<kanr hta1-119KE hta2-119KE z::Padh15-mCherry-atb2+<<natr
	SAK4117	h- ade6-M216 leu1 ura4-D18 sgo2+-GFP<<kanr hta1-119KR hta2-119KR z::Padh15-mCherry-atb2+<<natr
Figure 3E	SAK3695	h90 ade6 leu1 sgo1+-flag-GFP Prad21-slp1+<<kanr Prad21-cut23+<<kanr z::Padh15-mCherry-atb2+<<natr
	SAK3690	h90 ade6 leu1 hta1-S121A hta2-S121A sgo1+-flag-GFP Prad21-slp1+<<kanr Prad21-cut23+<<kanr z::Padh15-mcherry-atb2+<<natr
	SAK4087	h90 ade6 leu1 hta1-K119D hta2-K119D sgo1+-flag-GFP Prad21-slp1+<<kanr Prad21-cut23+<<kanr z::Padh15-mcherry-atb2+<<natr ura4-D18?
	SAK4091	h90 ade6 leu1 hta1-K119E hta2-K119E sgo1+-flag-GFP Prad21-slp1+<<kanr Prad21-cut23+<<kanr z::Padh15-mcherry-atb2+<<natr ura4-D18?
	SAK4094	h90 ade6 leu1 hta1-K119R hta2-K119R sgo1+-flag-GFP Prad21-slp1+<<kanr Prad21-cut23+<<kanr z::Padh15-mcherry-atb2+<<natr ura4-D18?

Figure S2A	sc221	MATa (hta1-htb1)⊿::LEU2+ (hta2-htb2)⊿::TRP1 his⊿200 trp1⊿63 lys2-128δ ura3-52 leu2⊿1 SGO1-GFP<<kanr SPC42-mCherry<<natr pJH55(HTA1WT-HTB1WT HIS3 CEN)
	sc222	MATa (hta1-htb1)⊿::LEU2+ (hta2-htb2)⊿::TRP1 his⊿200 trp1⊿63 lys2-128δ ura3-52 leu2⊿1 SGO1-GFP<<kanr SPC42-mCherry<<natr pJH55(HTA1S121A-HTB1WT HIS3 CEN)
	sc401	MATa (hta1-htb1)⊿::LEU2+ (hta2-htb2)⊿::TRP1 his⊿200 trp1⊿63 lys2-128δ ura3-52 leu2⊿1 SGO1-GFP<<kanr SPC42-mCherry<<natr pJH55(HTA1K119D-HTB1WT HIS3 CEN)
	sc403	MATa (hta1-htb1)⊿::LEU2+ (hta2-htb2)⊿::TRP1 his⊿200 trp1⊿63 lys2-128δ ura3-52 leu2⊿1 SGO1-GFP<<kanr SPC42-mCherry<<natr pJH55(HTA1K119E-HTB1WT HIS3 CEN)
	sc405	MATa (hta1-htb1)⊿::LEU2+ (hta2-htb2)⊿::TRP1 his⊿200 trp1⊿63 lys2-128δ ura3-52 leu2⊿1 SGO1-GFP<<kanr SPC42-mCherry<<natr pJH55(HTA1K119R-HTB1WT HIS3 CEN)
Figure S2B	sc335	MATalpha ade2-1 ura3-1 hta1-htb1::CgTRP1 hta2-htb2::hphMX4 CEN5::tetO2x112::HIS3 leu2::TetR-GFP::LEU2 bar1 RAD5 [pRS412 HTA1-HTB1(ADE2)]
	sc341	MATalpha ade2-1 ura3-1 hta1-htb1::CgTRP1 hta2-htb2::hphMX4 CEN5::tetO2x112::HIS3 leu2::TetR-GFP::LEU2 bar1 RAD5 [pRS412 HTA1S121A-HTB1(ADE2)]
	sc338	MATalpha ade2-1 ura3-1 hta1-htb1::CgTRP1 hta2-htb2::hphMX4 CEN5::tetO2x112::HIS3 leu2::TetR-GFP::LEU2 bar1 RAD5 [pRS412 HTA1K119D-HTB1(ADE2)]
	sc339	MATalpha ade2-1 ura3-1 hta1-htb1::CgTRP1 hta2-htb2::hphMX4 CEN5::tetO2x112::HIS3 leu2::TetR-GFP::LEU2 bar1 RAD5 [pRS412 HTA1K119E-HTB1(ADE2)]
	sc336	MATalpha ade2-1 ura3-1 hta1-htb1::CgTRP1 hta2-htb2::hphMX4 CEN5::tetO2x112::HIS3 leu2::TetR-GFP::LEU2 bar1 RAD5 [pRS412 HTA1K119R-HTB1(ADE2)]
	sc340	MATalpha ade2-1 ura3-1 hta1-htb1::CgTRP1 hta2-htb2::hphMX4 CEN5::tetO2x112::HIS3 leu2::TetR-GFP::LEU2 bar1 RAD5 [pRS412 HTA1K119Q-HTB1(ADE2)]
Figure	SAK4109	h- sgo2+-GFP<<kanr z::Padh15-mCherry-atb2+<<natr

S3A, C	SAK4111	h- ade6 leu1 sgo2+-GFP<<kanr hta1-S121A hta2-S121A z::Padh15-mCherry-atb2+<<natr
	SAK4113	h- ade6-M210 leu1 ura4-D18 sgo2+-GFP<<kanr hta1-119KD hta2-119KD z::Padh15-mCherry-atb2+<<natr
	SAK4115	h+ ade6-M216 leu1 ura4-D18? sgo2+-GFP<<kanr hta1-119KE hta2-119KE z::Padh15-mCherry-atb2+<<natr
	SAK4117	h- ade6-M216 leu1 ura4-D18 sgo2+-GFP<<kanr hta1-119KR hta2-119KR z::Padh15-mCherry-atb2+<<natr
Figure	SAK177	h+ leu1 cen2+-GFP<<kanr<<ura4+
S3B	SAK109	h+ leu1 ura4-D18 cen2+-GFP<<kanr<<ura4+ hta1-S121A hta2-S121A
	SAK4076	h+ ade6-M210 leu1 ura4-D18 cen2+-GFP<<kanr<<ura4+ hta1-K119D hta2-K119D
	SAK4077	h+ ade6-M216 leu1 ura4-D18 cen2+-GFP<<kanr<<ura4+ hta1-K119E hta2-K119E
	SAK4080	h+ ade6-M216 leu1 ura4-D18 cen2+-GFP<<kanr<<ura4+ hta1-K119R hta2-K119R
Figure	SAK1	h+ ade6-M210 leu1 ura4-D18
S3D	SAK176	h- ade6-M216 leu1 cen2+-GFP<<kanr<<ura4+
	PM173	h+ ade6 leu1 hta1-S121A hta2-S121A
	PL858	h- leu1 cen2+-GFP<<kanr<<ura4+ hta1-S121A hta2-S121A
	SAK4006	h- ade6-M210 leu1 ura4-D18 hta1-K119D hta2-K119D
	SAK4076	h+ ade6-M210 leu1 ura4-D18? cen2+-GFP<<kanr<<ura4+ hta1-K119D hta2-K119D
	SAK4084	h- ade6-M210 leu1 ura4-D18 hta1-K119E hta2-K119E
	SAK4085	h+ ade6-M210 leu1 ura4-D18? cen2+-GFP<<kanr<<ura4+ hta1-K119E hta2-K119E
